# Biocompatible Glycopolymer-PLA Amphiphilic Hybrid Block Copolymers with Unique Self-Assembly, Uptake, and Degradation Properties

**DOI:** 10.1021/acs.biomac.4c00885

**Published:** 2024-09-14

**Authors:** Kevin A. Green, Anuja S. Kulkarni, Penelope E. Jankoski, Thomas B. Newton, Blaine Derbigny, Tristan D. Clemons, Davita L. Watkins, Sarah E. Morgan

**Affiliations:** † School of Polymer Science and Engineering, The University of Southern Mississippi, Hattiesburg, Mississippi, Hattiesburg 39406, United States; ‡ Department of Chemistry & Biochemistry, The Ohio State University, Columbus, Ohio 43210, United States; § William G. Lowrie Department of Chemical and Biomolecular Engineering, The Ohio State University, 151 W Woodruff Avenue, Columbus, Ohio 43210, United States

## Abstract

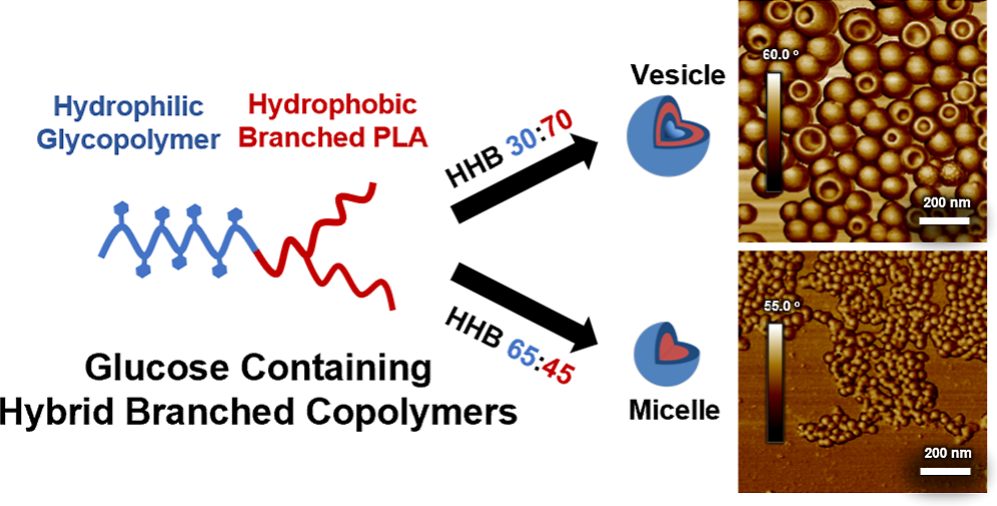

The self-assembly of Janus-type amphiphilic hybrid block copolymers composed of hydrophilic/hydrophobic layers has shown promise for drug encapsulation and delivery. Saccharides have previously been incorporated to improve the biocompatibility of self-assembled structures; however, glycopolymer block copolymers have been less explored, and their structure–property relationships are not well understood. In this study, novel glycopolymer-branched poly(lactic acid) (PLA) block copolymers were synthesized via thiol–ene coupling and their composition-dependent morphologies were elucidated. Stability as a function of pH, dye uptake capabilities, and cytotoxicity were evaluated. Systems with a hydrophilic weight ratio of 30% were found to produce bilayer nanoparticles, while systems with a hydrophilic weight ratio of 60% form micelles upon self-assembly in aqueous media. Regardless of composition and morphology, all systems exhibited uptake of both hydrophobic (curcumin, DL % from 4.25 to 11.55) and hydrophilic (methyl orange, DL % from 4.08 to 5.88) dye molecules with release profiles dependent on composition. Furthermore, all of the nanoparticles exhibited low cytotoxicity, confirming their potential for biomedical applications.

## Introduction

Polymeric nanoparticles (PNs), with their high processability and a wide range of available structural features, have emerged as a promising approach for therapeutic delivery. PNs are formed through the self-assembly of polymers like polyesters, poly alkyl-cyanoacrylates, or amphiphilic block copolymers.^1^ Amphiphilic hybrid block copolymers (HBCs) are a unique class of amphiphilic, branched, asymmetrical macromolecular structures similar to Janus dendrimers (JDs) that are capable of self-assembling into various nanostructures, including micelles and bilayer vesicles, dependent on their building blocks and compositions.^2−8^ These nanostructures benefit from dendrimer-like, branched architectures that permit entrapment of drugs via three different mechanisms: (1) entrapment within void spaces, dependent on the size of the drug; (2) secondary or electrostatic bonding of the drug to dendrimer branch points; and (3) charge–charge interactions at the dendrimer surface if the drug and dendrimer are oppositely charged.^9,10^ Initially, Janus dendrimersomes (JDSs) depended on cationic moieties to aid in cell transfection, which proved toxic at higher concentrations and dendrimer generations.^11^ Alternatively, polyethylene glycol (PEG) can be used as the hydrophilic block to reduce toxicity.^12,13^ However, both PEG and its derivatives have also been implicated in patient sensitivities and may reduce cell internalization.^14−16^

Researchers have previously reduced JDS cytotoxicity and enhanced dendrimersome capabilities by incorporating saccharide structures on the dendrimer surface.^5,17,18^ Using facile copper-catalyzed click coupling chemistries, Percec and co-workers attached saccharide groups to the PEG blocks within JD.^5^ These Janus glycodendrimers self-assembled into Janus glycodendrimersomes (JGDSs), which mimicked various membrane-containing organelles.^5,19−21^ The incorporation of saccharide structures imparts a multivalent nature to JGDS, with abundant free surface saccharides providing numerous attachment sites for receptor proteins, thus replicating the glycocluster effect found in natural systems.^18^ The saccharide surface-modified JGDS exhibited increased hydrophilicity, enhanced bioactivity (evidenced by increased saccharide–lectin interactions), and reduced nanoparticle cytotoxicity.^6,7^

Glycopolymers, with their biocompatibility, low cytotoxicity, high degree of hydrophilicity, and cellular recognition capabilities, are promising alternatives to traditional hydrophilic blocks that can be tailored to address specific needs.^22,23^ Synthetic glycopolymers with pendant saccharides possess a higher number of free hydroxyl groups compared with naturally occurring polysaccharides, where some hydroxyl groups are involved in backbone glycosidic linkages. This abundance of free hydroxyl groups in synthetic glycopolymers enhances water solubility and increases their propensity to aggregate and/or associate with other molecules in solution.^23^ While saccharides have been decorated on dendrimersome surfaces, the use of glycopolymers as the hydrophilic block of the amphiphilic copolymer has been less explored, and the structure–property relationships of these systems are not well understood.^24−26^

To prepare block copolymers of varying hydrophobicity, blocks can be synthesized sequentially or separately.^27^ Synthesizing each block separately provides greater control over molecular weight, dispersity, and copolymer composition, which significantly influences the self-assembled morphology of the nanostructures. Consequently, polymer–polymer coupling has become a popular method for synthesizing complex polymer architectures.^28^ Utilizing facile click chemistries that are straightforward and require mild reaction conditions, researchers have produced complex architectures using reactive chain ends.^29−31^ Thiol–ene, thiol–yne, and azide–alkyne click chemistries are particularly promising for small-molecule coupling reactions due to their high yields (65 to 90%), overall selectivity, and reduced number of side products.^31−33^ However, achieving high yields in complex polymer–polymer coupling reactions remains challenging due to unwanted steric interactions and the inherently low concentration of reactive chain ends.^33^

Block copolymer self-assembly morphology is determined by block ratios.^34^ Similar to surfactants, HBC nanostructure morphologies are also controlled by the hydrophilic/hydrophobic balance (HHB) of the two coupled blocks.^35−37^ In aqueous media, amphiphilic HBCs spontaneously self-assemble into various nanostructures to minimize the system’s overall free energy.^38,39^ Core–shell micellar morphologies have been extensively studied as drug delivery vehicles due to their amphiphilic core–corona structure, featuring a hydrophobic core and a hydrophilic corona. The hydrophilic shell stabilizes the structure and serves as a “stealth layer” for penetration into organelles, while the hydrophobic core entraps hydrophobic small molecules, such as dyes and drugs.^35^ These structures form only when the hydrophilic block weight fraction is equal to or longer than that of the hydrophobic block. Vesicular structures offer improved mechanical stability for nanomedicines compared to micellar structures.^35,40^ Vesicular structures (liposomes, polymersomes, and dendrimersomes) are bilayer nanoparticles with exterior and interior hydrophilic shells and a hydrophobic midlayer. Polymeric vesicles composed of biodegradable block copolymers exhibit increased stabilities, extended circulation times, and slower payload release compared to polymeric micelles.^35,40^

**Scheme 1:**
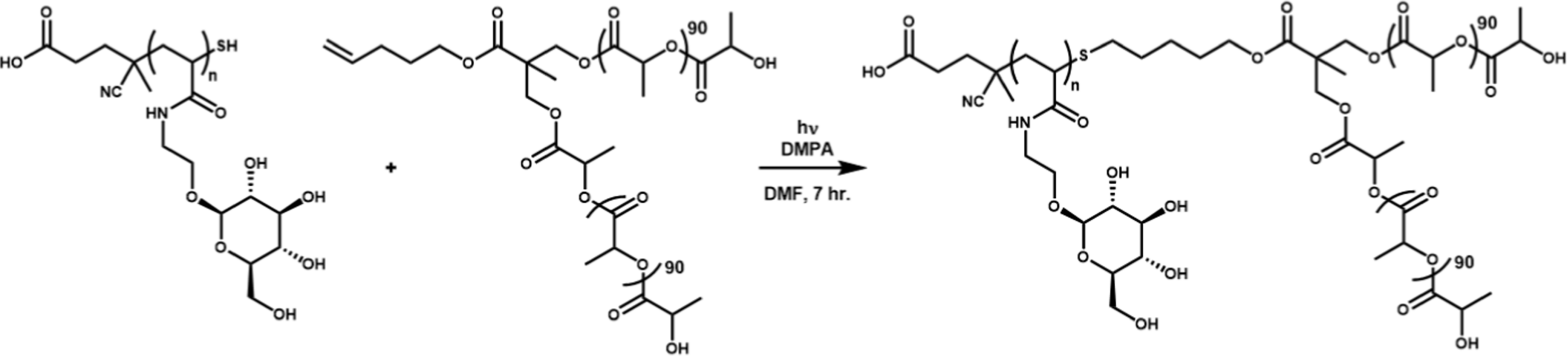
General Synthesis of HBC Obtained by the Photoinitiated Thiol–Ene Coupling of an Acrylamide Block with Glucose Pendant Groups (*n* = 23, 27, 95, or 162) and a Branched PLA Block

In this work, a library of HBCs composed of polyacrylamide-based glycopolymers with β-d-glucose pendant groups (hydrophilic block) and branched poly(lactic acid) (PLA) (hydrophobic block) were synthesized at varying HHB to determine the effect of the hydrophilic ratio on self-assembled morphology and dye uptake. Our approach introduced a novel synthesis of linear glycopolymer-branched PLA copolymers prepared through thiol–ene coupling, as shown in Scheme 1. The HBCs were used to form nanoparticles in aqueous media by nanoprecipitation, and the resulting structures were characterized via atomic force microscopy (AFM), transmission electron microscopy (TEM), and dynamic light scattering (DLS). To verify the potential of these materials as delivery vehicles, the encapsulation of both hydrophobic (curcumin) and hydrophilic [methyl orange (MO)] molecules, as model fluorophores, was demonstrated. Finally, cellular viability was evaluated using human embryonic kidney (HEK) cells with a lactate dehydrogenase (LDH) assay and LIVE/DEAD cell staining, and results were compared with those of hydrophilic blocks traditionally used in biomedical applications.

## Experimental Methods

### Materials

*N*-Hydroxyethyl acrylamide (97%), silver trifluoromethanesulfonate (AgOTf, ≥ 99%), acetobromo-α-d-glucose (AcBrGlc, ≥ 95%), 4 Å molecular sieves (powdered), anhydrous sodium sulfate (≥99%), 4′-azobis(4-cyanovaleric acid) (V-501), trimesic acid (95%), anhydrous dimethyl sulfoxide (≥99.9%), sodium methoxide solution (25 wt % in methanol), tris hydrochloride (tris HCl, ≥ 99%), tris base (≥99%), 4-penten-1-ol, 2-(hydroxymethyl)-2-methylpropanoic acid, dimethyl amino pyridine (DMAP), para toluene sulfonic acid, 1,8-diazabicyclo [5.4.0] undec-7-ene (DBU), DOWEX 50 W XB, Amberlyst A 21 free base, 2,2-dimethoxy-2-phenylacetophenone (DMPA, ≥ 99%), curcumin, MO, and sodium hydroxide solution (NaOH, 5.0N) were purchased from Sigma-Aldrich. Dichloromethane (DCM), ethyl acetate, hexane, methanol, tetrahydrofuran, concentrated hydrochloric acid, *N*,*N*-dimethylformamide (DMF), HPLC water, sodium azide (≥99%), and pH reference buffers (pHs of 4.00, 7.00, and 10.00) were purchased from Fisher Scientific. The chain transfer agent, 4-cyano-4-(((ethylthio)carbonothioyl)thio)pentanoic acid (CEP), was purchased from AmBeed. Chloroform-*d* (D, 99.9%), deuterium oxide (D, 99.9%), dimethyl sulfoxide-*d*_6_ (D, 99.9%), and *N,N*-dimethylformamide-*d*_7_ (D, 99.9%) were purchased from Cambridge Isotope Laboratories, Inc. Dicyclohexyl carbodiimide (DCC) was procured from the Tokyo Chemical Industry (TCI). Uranyl acetate was purchased from Electron Microscopy Sciences. The LIVE/DEAD Cell Imaging Kit (488/570, Invitrogen) was obtained from Thermo Scientific.

### Synthesis of Glycomonomer

The acetyl-protected glucose pendant acrylamide monomer, 2′-acrylamidoethyl-2,3,4,6-tetra-O-acetyl-β-d-glucopyranoside (AcGlcEAm), was synthesized following previous literature procedures (Scheme S1 and Figure S1).^41−43^

### RAFT Polymerization of Glycomonomer

Glucose pendant glycopolymers (pGlcEAm) were synthesized following a previously reported procedure, with the exception of collecting the polymer at 60% rather than 70% conversion (Scheme S2 and Figures S2, S3, and S4).^43^

### Branched Polylactic Acid Synthesis

The PLA was synthesized through a 4-step process. The precursor, 2,2,5-trimethyl-1,3-dioxane-5-carboxylic acid (**1**), was synthesized following a previously reported procedure, Scheme S3.^36^

**Scheme 2:**
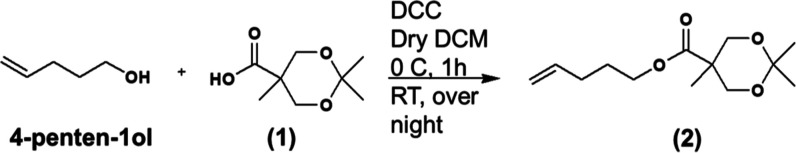
Synthesis of 4-Penten-1-acetonide

4-Penten-1-acetonide (**2**) was synthesized as outlined in Scheme 2. 4-Penten-1-ol (0.6 g, 5.8 mmol), **1** (1.9 g, 11.61 mmol), and DMAP (0.44 g, 3.63 mmol) were stirred in anhydrous DCM (30 mL) at 0 °C under argon. DCC (1.2 g, 5.8 mmol) was added to the reaction flask under argon. The reaction mixture was stirred overnight at room temperature. The progression of the reaction was monitored by TLC. Once the reaction reached completion, the reaction mixture was worked up with 0.5 N HCl and concentrated, and the product was then purified via column chromatography using 100% hexanes (0.85 g, 50% yield). ^1^H NMR (400 MHz, CDCl_3_, δ): [ppm] 5.80 (m, 1H), 5.02 (m, 2H), 4.18 (t, 2H, *J =* 11 Hz), 4.17 (d, 2H, *J* = 7 Hz), 3.64 (d, 2H, *J =* 12 Hz), 2.14 (m, 2H), 1.76 (m, 2H), 1.40 (m, 6H), 1.19 (s, 3H).

**Scheme 3:**
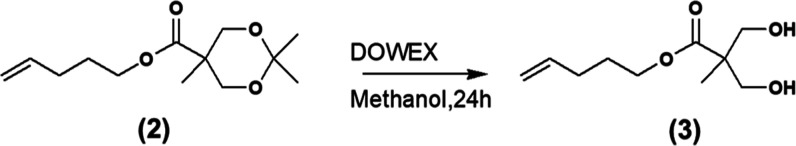
Synthesis of 4-Penten-2-hydroxyl

4-Penten-2-hydroxyl (**3**) was synthesized as outlined in Scheme 3. **2** (10.0 g, 35.7 mmol) was dissolved in methanol (200 mL), and 24 g of DOWEX 50W-X2 resin was added to the reaction vessel. The solution was stirred at room temperature for 24 h, and the completion of the reaction was confirmed by ^1^H NMR. The resin was filtered off and thoroughly washed with methanol. The filtrate was concentrated in a vacuum to give **3** (7.56 g, 89%) as a colorless oil. ^1^H NMR (400 MHz, CDCl_3_, δ): [ppm] 5.73 (m, 1H), 4.96 (m, 2H), 4.12 (t, 2H, *J* = 8 Hz), 3.85 (d, 2H, *J* = 12 Hz), 3.65 (d, 2H, *J = 12* Hz), 2.08 (m, 2H), 1.72 (m, 2H), 0.99 (s, 3H).

**Scheme 4:**
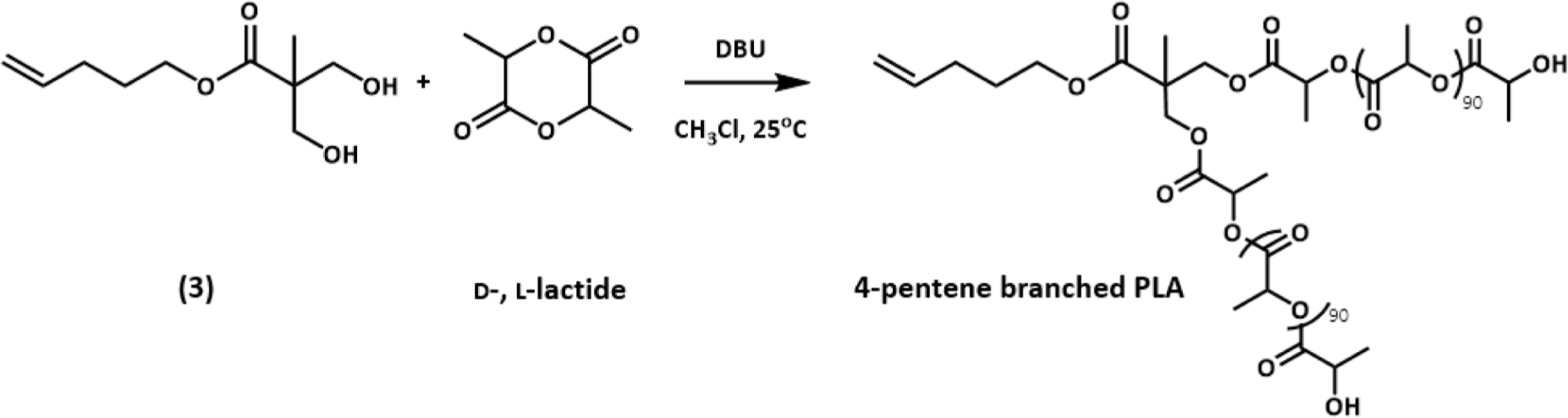
Polymerization of D- and l-Lactide Using 4-Penten-2-hydroxyl by Ring Opening Polymerization (ROP)

Branched PLA was synthesized as outlined in Scheme 4. The monomer (**3**) (0.050 g, 0.247 mmol) and D- and l-lactide (3.35 g, 0.24 mmol) were added in a round-bottom flask (RBF) sealed with a rubber septum and diluted with anhydrous chloroform. The RBF was subjected to three freeze–thaw cycles before being transferred to the glovebox. DBU was purified by maintaining it under vacuum for 7 days and stored under nitrogen before transferring it to the glovebox. DBU (0.188 g, 1.236 mmol) was added in another RBF sealed with a rubber septum (subjected to freeze–thaw cycles) maintained in the glovebox and diluted with anhydrous chloroform. DBU solution was added dropwise to the monomer solution under argon. The reaction was allowed to stir at room temperature for 4 h and quenched by adding benzoic acid (0.226 g, 1.85 mmol). The reaction was further allowed to stir for another 1 h. The resulting viscous liquid was purified from methanol/hexane/ether mixture (1:15:5). The polymer obtained was dissolved in DCM, reprecipitating three times using the above-mentioned mixture. The resulting polymer was finally dried under vacuum for 48 h.

### Photocoupling Synthesis of Amphiphilic HBCs

As outlined in Scheme 1, the HBCs were synthesized through a thiol–ene photocoupling reaction adapted from the literature.^31−33^ Glycopolymer was added in excess to PLA (1.5:1 molar ratio) in order to ensure completion of the reaction, while the ratio of PLA to the photoinitiator (DMPA) was kept constant at 1:1. In order to completely solubilize both materials, glycopolymer (350 mg, 0.0075 M) was first loaded into a 20 mL scintillation vial equipped with a magnetic stirring bar, dissolved in the total reaction volume of DMF (3.91 mL), and allowed to stir overnight. The following day, PLA (250 mg, 0.005 M) and trimesic acid (2.05 mg, 0.0025 M), the internal standard, were added and stirred at 40 °C until fully dissolved. At this point, the photoinitiator, DMPA (5.02 mg, 0.005 M), was added. The reaction vessel was then covered with foil and quickly transferred to a photochemical reactor (Rayonet) equipped with a cooling fan and 16 UV lamps (λ = 350 nm), where the reaction was allowed to proceed. Conversion was monitored using ^1^H NMR, comparing the disappearance of vinyl protons to the internal standard. After 7 h, a typical reaction showed that coupling conversion of 38% was achieved. Reactions were then kept in dark conditions for 12 h, and the HBC was purified by precipitation into DI water and filtered to remove unreacted glycopolymer. The solids were then washed with cold THF to remove unreacted PLA. Washed products were then placed under N_2_ to remove residual THF and lyophilized to isolate the HBC as a white powder.

### Nanoprecipitation Self-Assembly

HBCs with HHBs of 31:69, 38:62, 66:34, and 76:24 (glycopolymer: PLA) were formed into nanoparticles via nanoprecipitation following published procedures.^35,37,44^ HBC samples (1 mg) were added to glass vials, THF (200 μL) was added, and the sample was vortexed until fully dissolved. The resulting solution (organic phase) was then added dropwise into a second vial containing DI water (2 mL) while vigorously stirring. The solution was then covered and allowed to equilibrate overnight, allowing residual THF to evaporate. Nanoparticle concentration was maintained at 0.5 mg mL^–1^ for characterization. To further test nanoparticle stability with changes in pH, the nanoprecipitation procedure was repeated at varying pH conditions (1, 3, 7, 10, and 12) adjusted with either HCl or NaOH.

### Nuclear Magnetic Resonance Spectroscopy

^1^H NMR spectroscopy was performed using either a 400 or 600 MHz Bruker AVANCE III spectrometer (TopSpin 3.1p17 software). Monomer spectra were acquired with 64 coadded scans and a delay time of 5 s. Polymer and HBC spectra were acquired with 64 coadded scans and a delay time of 2 s. All spectra were obtained by using the appropriate deuterated solvents (CDCl_3_, DMSO-*d*_6_, D_2_O, or DMF-*d*_7_) and were processed and analyzed by using MNova software.

### Ultraviolet Visible Spectroscopy

Cleavage of the trithiocarbonate end group was verified using a multimode microplate reader (BioTek Synergy H1, Agilent Technologies Inc.) by obtaining the absorbance at 310 nm. Each measurement was conducted in DMSO using a sample volume of 200 μL and a sample concentration of 2.5 mg mL^–1^ at 25 °C. Absorbance data was analyzed using BioTek Gen6 data analysis software.

### Gel Permeation Chromatography with Multiangle Laser Light Scattering

Glycopolymer weight- and number-average molecular weights were determined using aqueous size exclusion chromatography with multiangle laser light scattering (ASEC-MALLS) on an Agilent 1260 Infinity II LC system equipped with a PL Aquagel–OH 30 column (particle size 8 μm), a DAWN HELEOS-II light scattering detector (λ = 633 nm, Wyatt Technology Inc.), and an Optilab T-rEX refractometer (Wyatt Technology Inc.). Tris buffer of pH 8.0 with 0.01% (w/v) NaN_3_ was used as the eluent at a flow rate of 0.5 mL min^–1^ with a sample concentration of 20 mg mL^–1^ and an injection volume of 100 μL. The polymer refractive index increments (d*n*/d*c*) were determined using an offline refractometer (AR200 Refractometer, Reichert) at 25 °C. Wyatt ASTRA SEC/LS software (version 7.1.4.8) was used to determine the number-average molecular weight (*M*_*n*_), weight-average molecular weight (*M*_w_), and polymer dispersity (*D̵*).

PLA molecular weights were determined using GPC-MALLS on a Waters Alliance 2695 separation module, an online MALLS detector fitted with a gallium arsenide laser, an interferometric refractometer operating at 35 °C and 685 nm, and two Agilent PLgel-mixed D columns (pore size range of 50–103 Å, 3 μm bead size). Distilled THF served as the mobile phase and was delivered at a flow rate of 1.0 mL min^–1^. The absolute molecular weights were determined by MALLS using a d*n*/d*c* calculated from the refractive index detector response and assuming 100% mass recovery from the columns.

### Atomic Force Microscopy

The nanoparticle morphology was characterized by AFM in Peak-Force Tapping mode (Dimension Icon AFM, Bruker) using an RTESPA-300 (*f*_0_ = 300 kHz, *k* = 40 N m^–1^, Bruker) probe. Samples were prepared by drop casting 150 μL of the nanoparticle solution onto a freshly cleaved mica substrate, waiting 30 min, wicking away excess solution, and allowing the samples to dry ambiently. Images were analyzed using NanoScope Analysis 3.00 software.

### Transmission Electron Microscopy

Samples were prepared for TEM by drop casting an aqueous solution of HBC samples (0.5 mg mL^–1^, DI water, 4 μL) onto 200-mesh copper grids with continuous carbon film (Electron Microscopy Sciences, Hatfield, PA, USA). After air drying, samples were incubated with 1% uranyl acetate (30 s), followed by wick removal of the solution with filter paper to enhance contrast during imaging. TEM was performed on a JEOL JEM-1400 microscope at a 120 kV accelerating voltage.

### Dynamic Light Scattering

Intensity average nanoparticle size measurements were conducted using a Zetasizer Nano ZS (Malvern Instrument) at 25 °C. The nanoparticle concentration was 0.5 mg mL^–1^, all measurements were performed in triplicate, and the data was analyzed using the provided Zetasizer software.

### Encapsulation Studies

Curcumin and MO dyes were used separately to load into nanoparticles following literature procedures with modifications.^35^ The chemical structures and absorbance profiles for each dye are included in the Supporting Information (Figure S15).

For the hydrophobic dye, curcumin (1 mg) and HBC (1 mg) were dissolved in THF (200 μL). This organic phase was added dropwise to DI water (2 mL) to prepare the final nanoparticle concentration (0.5 mg mL^–1^). Solutions were then covered with perforated aluminum foil, and the THF was allowed to evaporate overnight before any dye that was not encapsulated and had precipitated from solution. Solutions were then lyophilized, and the resulting powder was redissolved in THF (2 mL). In order to construct a calibration curve for free curcumin, solutions of dye in THF were prepared with concentrations ranging from 0 to 0.1 mg mL^–1^, and the absorbance values were determined using a microplate reader to measure absorbance at 430 nm. The dye loading efficiency (DL %) and encapsulation efficiency (EE %) were determined for each nanoparticle composition and dye species using eqs 1 and 2
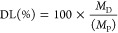
1
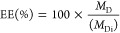
2with *M*_D_ = mass of dye in the nanoparticle, *M*_P_ = mass of HBC, and *M*_Di_ = mass of dye initially added during nanoprecipitation.^35^

For hydrophilic dyes (MO), a stock solution in DI water was prepared at 1 mg mL^–1^ to act as the aqueous phase, and 2 mg of the HBC was dissolved in THF (200 μL) to act as the organic phase. Similar to the standard nanoprecipitation procedure, the organic phase was added dropwise to the aqueous phase while stirring to prepare the final nanoparticle concentration (0.5 mg mL^–1^).^44^ Solutions were then transferred to 2 kDa MWCO dialysis tubing and dialyzed for 3 days until unencapsulated dye completely escaped, as determined by testing the outer solution with UV–vis (absorbance values of 464 nm for MO) until no signal from the dye was detected. The amount of encapsulated dye was calculated by using absorbance values compared with a standard calibration curve. To construct the calibration curve, solutions of hydrophilic dye dissolved in DI water were prepared with concentrations ranging from 0 to 0.1 mg mL^–1^, and a microplate reader was used to measure absorbance at 464 nm. The encapsulated dye, DL (%), and EE (%) were then calculated by using eqs 1 and 2.

### Cell Viability

HEK cells (HEK293, ATCC) were maintained in Dulbecco’s modified Eagle’s medium (DMEM) supplemented with 10% fetal bovine serum and 1% penicillin–streptomycin at 37 °C and 5% CO_2_. Cells at 90% confluence were trypsinized [0.25% trypsin ethylenediaminetetraacetic acid (EDTA), 5 min] to dissociate cells, collected into a falcon tube, and centrifuged at 5000 rpm for 5 min to form a pellet. HEK293 cells were resuspended in supplemented DMEM and counted using a hemocytometer to determine the cell concentration. Cells were diluted with additional media to a working concentration of 1 × 10^5^ cells mL^–1^ and seeded in a 96-well plate (200 μL per well). Seeded cells were left to adhere for 24 h in an incubator at 37 °C and 5% CO_2_. After 24 h, nanoparticle stock solutions were added to the wells to achieve the desired final concentration with nuclease free water used as a negative control, and Triton X-100 was used as positive control for 100% cytotoxicity (i.e., maximum LDH release). Plates were then incubated for 24 h at 37 °C and 5% CO_2_. Cytotoxicity was evaluated using the CyQUANT LDH Kit (Invitrogen) following the manufacturer’s protocols and was calculated using eq 3. A microplate reader was used to assess the absorbance at 490 nm with a reference wavelength of 690 nm
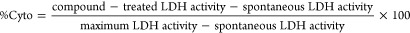
3
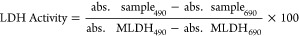
4

### LIVE/DEAD Imaging

The LIVE/DEAD Cell Imaging Kit (Invitrogen) was used to assess cell cytotoxicity at the highest concentration of nanoparticle treatment following the manufacturer’s protocols. Cells were plated and treated by following the cytotoxicity protocol. Following 24 h incubation with nanoparticle treatments, 180 μL of media was removed from each well, leaving 20 μL and an equal volume of freshly prepared LIVE/DEAD (calcein-AM and BOBO-3) stock solution added to each well. This was left to incubate for 15 min at room temperature in the dark. Five representative images were then collected per treatment using a Leica DM IL LED Fluo SE inverted fluorescent microscope.

## Results and Discussion

### Synthesis of Amphiphilic HBCs

A series of HBCs were synthesized via thiol–ene coupling of well-defined linear glycopolymers of varying molecular weights with a branched PLA of constant molecular weight (Scheme 1). Glycopolymers and PLA were prepared prior to the click reaction. Glycopolymers were synthesized via RAFT following previously published methods as described in the Experimental Section. Molecular weights of the linear polymers were targeted to achieve HHB of 30:70, 40:60, 65:35, and 75:25 when coupled with the branched PLA of constant molecular weight (*M*_*n*_ = 13.8 kDa) (Scheme S2).

To determine the appropriate conditions, glycopolymers were prepared in triplicate and reaction kinetics were evaluated. After the initial induction period, about 60 to 90 min, pseudo-first-order polymerization kinetics were observed for all target molecular weights (Figure S2). A loss of control of the polymerization is evident at monomer conversions above 60%; therefore, target monomer conversions were kept at ρ = 0.6 to limit the risk of polydisperse systems. Initial polymerization attempts yielded molecular weights 1.2–1.5× higher than expected, which has been reported for acrylamides polymerized under similar conditions by both Thomas et al. and our group.^43,45^ This behavior was attributed to a CTA efficiency of less than 100%. To correct this issue, reaction times were adjusted to achieve target molecular weights to match the desired HHB.

Target conversion was achieved for all systems at reaction times between 200 and 240 min. Following successful synthesis, hydroxyl group deprotection and cleavage of the trithiocarbonate end group, residual from the CTA, were accompanied by a loss in yellow color and verified by both ^1^H NMR spectroscopy, Figure S3, and UV–vis absorbance spectra, Figure S4. Acetyl-protecting group removal is verified by the disappearance of the methyl group peaks at 2.20 ppm (D_2_O) or 2.00 ppm (DMSO-*d*_6_) in ^1^H NMR spectra and a downfield shift of the aromatic proton peaks from 4.30 to 4.50 ppm (DMSO-*d*_6_), Figure S3. A decrease/disappearance in the UV–vis absorption at 310 nm was observed, indicative of successful cleavage of the trithiocarbonate end group, Figure S4.^46^

**Figure 1:**
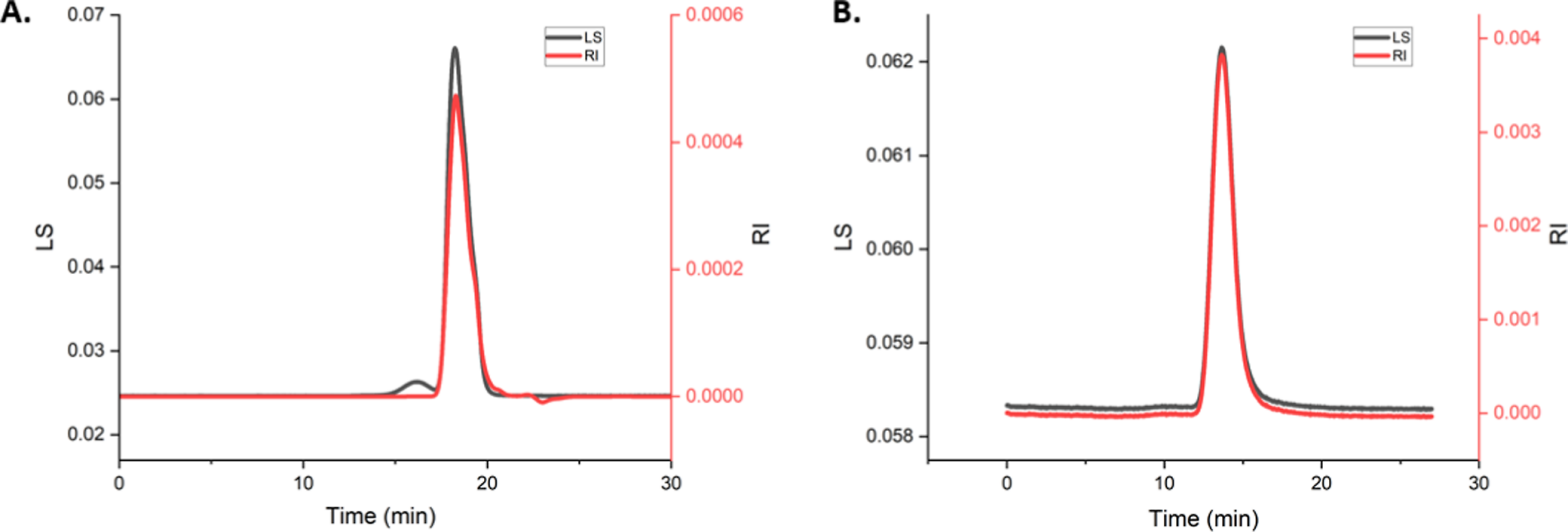
GPC traces. (A) ASEC-MALLS trace of pGlcEAm 21 determined in Tris buffer (pH 8) with 0.01% (w/v) NaN_3_. (B) GPC-MALLS trace of branched PLA determined in distilled THF.

Molecular weights and dispersity values collected from ASEC-MALLS traces of the glycopolymers are summarized in Table 1. A representative GPC trace of pGlcEAm 21 is shown in Figure 1A; others are provided in Figure S5. The glycopolymers cover a range of molecular weights (6.4 to 45 kDa), display narrow and symmetric chromatograms, and have low molecular weight distributions, *D̵* < 1.1. The high molecular weight shoulder present in light scattering spectra, but not in refractive index spectra, is likely associated with a low concentration of higher molecular weight aggregates, often seen in glycopolymer systems.^41−43,47,48^

**Table 1:** Composition, Conversion (ρ), Molecular Weight (*M*_*n*_), Dispersity (*D̵*), d*n*/d*c* Values, and Block Copolymer Naming Convention

polymer and target DP	target HHB	ρ^a^	*M*_*n*_^b^ (kDa)	*D̵*^b^	d*n*/d*c*^c^	calculated HHB	block copolymer naming
pGlcEAm 21	30:70	0.54	6.5	1.01	0.1546	31:69	Glc 31:69
pGlcEAm 49	40:60	0.68	7.7	1.04	0.1556	38:62	Glc 38:62
pGlcEAm 115	70:30	0.77	26.6	1.01	0.1570	66:34	Glc 66:34
pGlcEAm 131	80:20	0.61	45.1	1.01	0.1594	76:24	Glc 76:24

a Conversion determined by 400 MHz ^1^H NMR spectroscopy in DMSO, relaxation delay = 5 s.

b Determined using ASEC-MALLS in Tris buffer (pH = 8.0) with a PL aquagel MIXED–OH column. Flow rate of 0.5 mL min^–1^ and sample concentrations of 20 mg mL^–1^.

c Glycopolymer d*n*/d*c* values determined using an offline refractometer at 25 °C.

The branched PLA block was synthesized with a 4-penten-1-ol end group, chosen for use in thiol–ene click coupling. The acetonide-protected bis-MPA was used to modify 4-penten-1-ol to achieve two hydroxyl terminated initiators, resulting in the divergent growth of the polymer via DCC coupling. The hydroxyl groups of bis-MPA were first protected using DMP, confirmed by the appearance of acetonide peaks at 1.44 ppm (Figure S6). The protected bis-MPA was then conjugated to the 4-penten-1-ol using a DCC coupling reaction, confirmed by the shift in the CH_2_O peak of Bis-MPA from 3.69 and 4.17 ppm (Figure S7). The acetonide group deprotection was carried out by stirring the protected pentene derivative in methanol and acidic DOWEX-50W XB resin. The absence of the methyl groups at 1.40 ppm confirmed the deprotection (Figure S8). The deprotecting reactions resulted in good yields, and the coupled products purified by column chromatography were eluted with a gradient of hexane.

The synthetic approach utilized for the synthesis of branched PLA was the introduction of two lactide chains. A 4-pentene-1-ol modified with bis-MPA, containing two –OH focal points (**3**), was used as the initiator for the ROP of a mixture of D- and L-lactide monomer, resulting in a random branched polyester block. The hydrophobic wt % of the polylactide was kept constant (70%) regardless of the branching. DBU was used as a catalyst for this reaction as it has been widely reported for the ROP of lactides.^49^ The equivalent of the catalyst was maintained at 0.25 per hydroxyl group. Resonance peaks corresponding to the modified 4-pentene (multiplets at 5.78, 4.99, and 4.00 to 4.3 ppm) and for polylactide (br. peak at 5.13 ppm for –CH at and 1.56 ppm for –CH_3_, respectively, and the terminal –CH peak at 4.34 ppm) are observed in the ^1^H NMR spectrum, confirming the polymerization of the lactide in the presence of modified 4-pentene as the focal point (Figure S9). A narrow and symmetric chromatogram is observed by GPC-MALLS (Figure 1B), *M*_*n*_ = 13.80 kDa, *M*_w_ = 13.81 kDa, and *D̵* < 1.1.

Thiol–ene click photocoupling was chosen for three main reasons: (1) materials do not require additional postpolymerization modification other than the base-catalyzed deprotection, (2) copper contamination is avoided, and (3) photoinitiation reduces the risk of thermal degradation during reaction. Reaction conversion was determined through ^1^H NMR spectroscopy by comparing the PLA vinyl proton peaks (a quintet at 5.9 ppm in DMF-*d*_7_) to the internal standard, trimesic acid (8.8 ppm in DMF-*d*_7_). After 7 h, the conversion was calculated to be ∼38% (Figure S10). Polymer–polymer coupling reactions are known to have low conversion due to the low concentration of end groups and steric bulk from the polymer chain. Similar synthetic designs done by Fairbanks et al. reported a conversion of 21% when using allyl ethoxy as the vinyl moiety, DMSO as the solvent, and DMPA as the photoinitiator.^33^ Unreacted materials were recovered through a series of washes, and ^1^H NMR of the isolated HBC demonstrates successful coupling without any indication of degradation (Figure S11). Coupling was also verified by the formation of well-defined nanostructures after self-assembly. Naming conventions for the HBCs are given in Table 1. It was not possible to obtain the GPC trace of the block copolymer due to incompatibility of the amide structure with the column; however, the formation of stable nanostructures coupled with spectroscopy indicates successful coupling.

### Self-Assembly and Nanostructure Morphology

**Figure 2:**
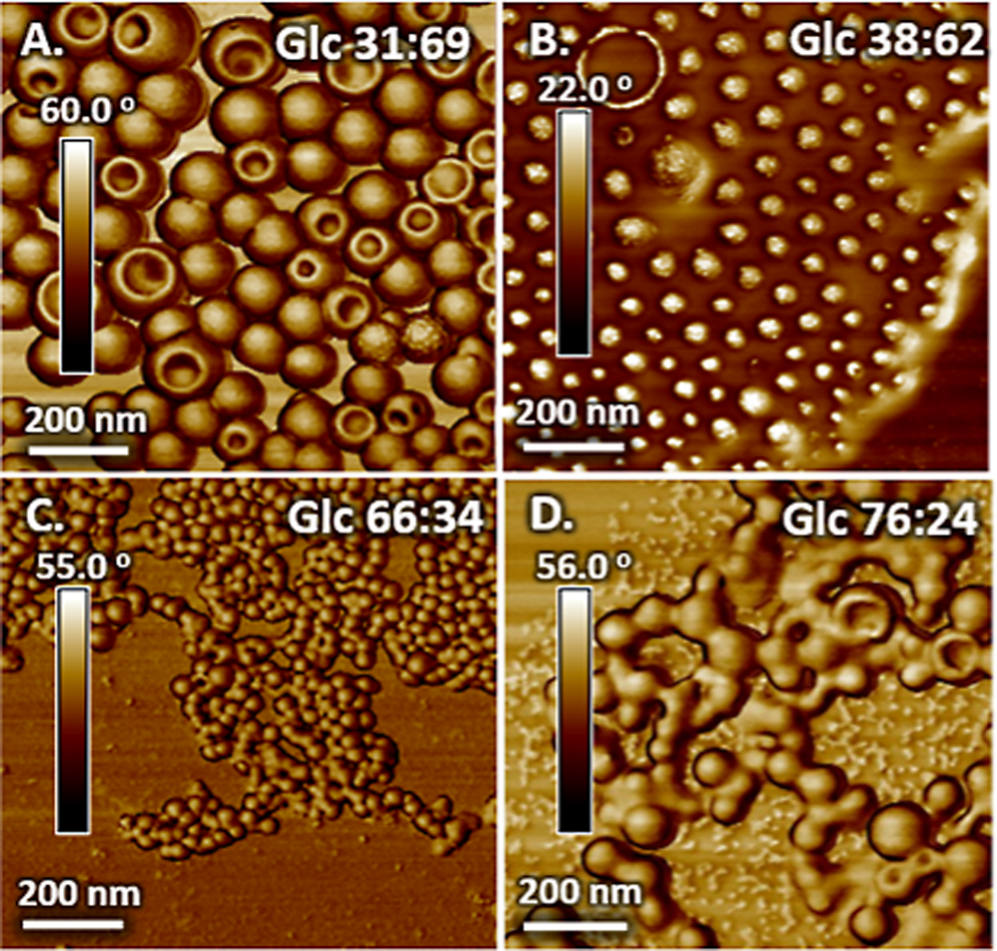
AFM phase images of (A) Glc 31:69, (B) Glc 38:62, (C) Glc 66:34, and (D) Glc 76:24. Scale bars are 200 nm.

A nanoprecipitation method, in which an organic solution of HBCs was added dropwise into DI water, was employed to allow self-assembly to form nanoparticles.^35,50^ Nanoparticle size and morphology were investigated using a combination of AFM (Figure 2), TEM (Figure S12), and intensity average DLS (Figure S13), with results summarized in Table 2.

**Table 2:** Average Diameter of Nanoparticles Formed in Water by HBCs as Measured by AFM, TEM, and DLS

sample	AFM (nm)	TEM (nm)	DLS peak (nm)
Glc 31:69	120 ± 15	110 ± 22	160 ± 50
Glc 38:62	47 ± 6.9, 180 ± 4.6	48 ± 10	220 ± 67
Glc 66:34	41 ± 5.7	40 ± 7.0	140 ± 46
Glc 76:24			

Diameters of the nanoparticles vary with copolymer composition and method of measurement. Those obtained from AFM and TEM images are consistent with average diameters of equivalent size (within experimental error). The Glc 31:69 block copolymer, with the highest hydrophobic content, yields diameters that are higher than those of the other HBCs. The size differences are indicative of vesicle formation for the lowest hydrophilic content (Glc 31:69) and micelles for the higher hydrophilic content (Glc 66:34) block copolymers, and consistent with expectations.^51^ Diameters measured with DLS are much larger, indicating the aggregation of the particles in solution. There is a wide distribution in the DLS particle sizes, and no statistically significant difference in the average diameter is observed. Aggregation is commonly observed in glycopolymers, attributed to inter/intramolecular hydrogen bonding and hydrophobic interactions.^22,42,52^ However, differences in overall aggregation may be attributed to the glycopolymer content in the HBCs or differences in the surface area to volume ratios of the self-assembled structures.

The morphology of the self-assembled structures depends on block copolymer composition.^34^ The Glc 31:69 copolymers self-assemble into spherical, bilayer, vesicles that are roughly 120 nm in diameter, Figure 2A. In AFM dry-state imaging, bilayer vesicles typically display a “donut shape” where height domains at the center point are much lower than at their outer edge.^34^ This has two main causes: (1) the vesicles may rupture, releasing their contained fluid, which offers structure and stability, or (2) the force induced by the AFM probe, even in soft tapping mode, causes deformation of the vesicles.^53−56^ Similar larger structures are observed in TEM, with a higher electron density outer shell indicative of bilayer structures (Figure S12A).

Self-assembled nanostructures formed from Glc 66:34, Figure 2C, are smaller and appear to be spherical, solid, core–shell micelles with an average diameter of 41 nm. Micellar structures are expected at equal or greater weight fractions of hydrophilic to hydrophobic blocks, ≥50:50.^56^

Glc 38:62 shows a mixture of bilayer vesicles and micelles, Figure 2B. This indicates a transitional range of HHBs that does not favor one structure formation over the other. A range of diameters is observed, with micelles measuring around 47 nm and vesicles around 180 nm. TEM images (Figure S12B) also show a mixture of micelles, aggregates of micelles, and larger features with higher electron density outer shells, indicating bilayer vesicles. Due to the difficulty of estimating diameters of particles within the aggregates, in Table 2, we have reported measured TEM diameters only for the observed individual micelles. Note that for this mixed system near the 50/50 HHB, DLS shows one broad distribution with a large average diameter indicative of a high degree of aggregation, while in AFM, it is possible to distinguish features with two distinct diameter ranges.

Samples prepared by the self-assembly of Glc 76:24 resulted in irregular features that lack structural stability, Figure 2D. This is attributed to an insufficient hydrophobic content to achieve stable nanostructures for this HBC, and this copolymer was not used for further studies.

At hydrophilic concentrations lower than 70%, our HBCs display morphological regimes similar to those of traditional amphiphilic block copolymers, where 30% hydrophilic block copolymers produce bilayer vesicles, and ≥50% hydrophilic block copolymers produce core–shell micelles.^34^ In this concentration regime, the high aggregation propensity of the glycopolymer blocks does not appear to affect the morphology or stability of the self-assembled HBC nanoparticles. However, at hydrophilic content >70%, unstable systems are formed that do not maintain a regular structure. This work highlights the tailorability of glycopolymer-PLA HBCs, targeting molecular weights to form different morphologies that are not impacted by the hydrophilic block’s propensity to self-associate.

### pH Effects on Nanoparticle Size and Stability

**Figure 3:**
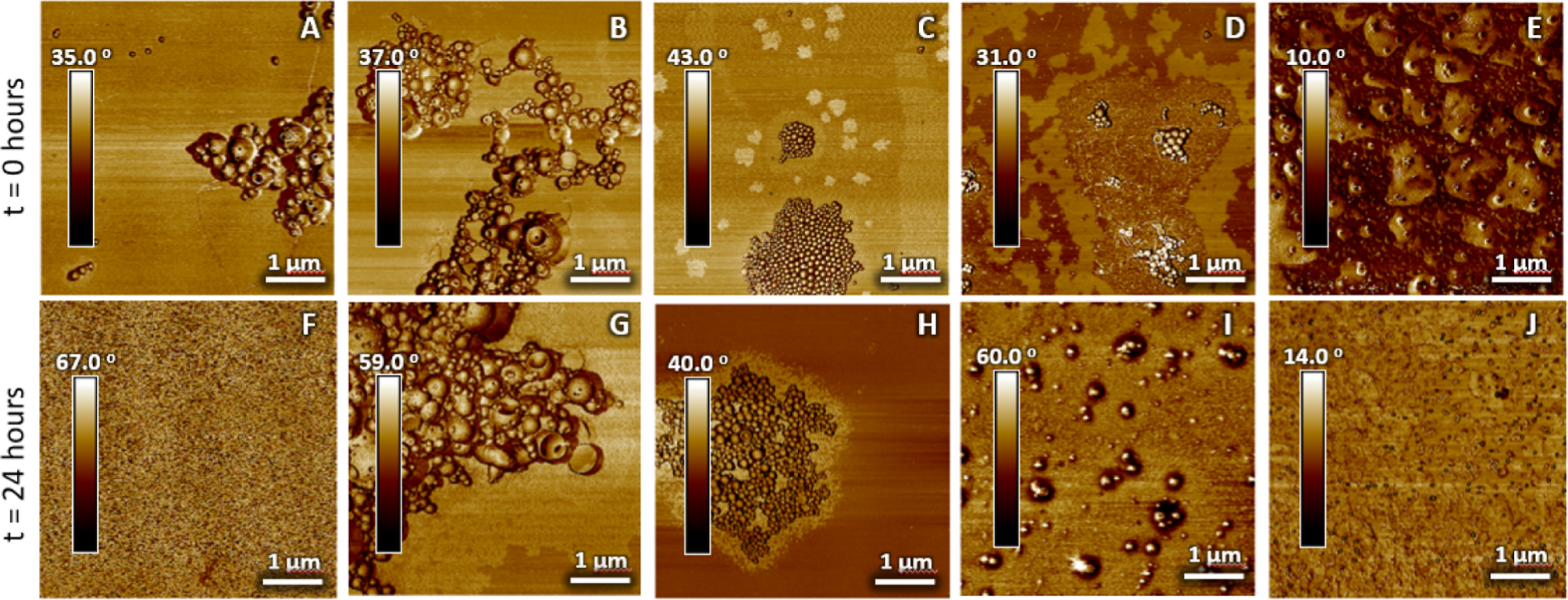
Self-assembly and nanostructure stability are dependent on the nanoprecipitation pH. (A–E) Representative AFM phase images of freshly prepared nanostructures of Glc38:62 at different pH values. (F–J) Representative AFM phase images of solutions after 24 h at different pH values. (A and F) Fresh and overnight phase images for pH 1; (B and G) fresh and overnight phase images for pH 3; (C and H) fresh and overnight phase images for pH 7; (D and I) fresh and overnight phase images for pH 10; and (E and J) fresh and overnight phase images for pH 12. Scale bars are 1 μm.

The effect of pH on nanoparticle self-assembly and stability over time is important for their use in biomedical applications. Physiological pH is in the range of 1–2 in the stomach, 6–7 in the intestines, and 7.35–7.45 in the blood, demonstrating the importance of understanding stability and responsiveness over a wide range of pH in the design of materials for oral and intravenous diagnostic and delivery applications.^57−59^ A series of samples were prepared by nanoprecipitation of Glc 38:62 (the sample with mixed morphology) in aqueous solution of varying pH (pH = 1–12) and morphological changes were studied after 24 h incubation. The effect of pH on the resultant nanostructure morphology is seen in Figure 3. Glc 38:62 nanoparticles freshly produced under acidic conditions display increased hydrodynamic diameters compared to those produced at neutral conditions, from 47 and 180 nm at pH = 7 to 77.6 and 425 nm at pH = 1, and 103 and 438 nm at pH = 3, respectively (Figure 3A, B, and C). This indicates that highly acidic conditions promote higher levels of aggregation for glucose-containing HBCs, which in turn form larger nanostructures. We attribute this to an increase in aggregation number, as there is no evidence of change in morphology, such as a transition from micelles to vesicles or vesicles to tubules.^60−64^ Nanoparticles produced under basic conditions (Figure 3D, E) maintain hydrodynamic diameters similar to those of nanoparticles formed in neutral environments: 63.7 and 138 nm at pH = 10 and 69.3 and 145 nm at pH = 12, respectively.

Nanostructures formed in basic environments display consistent homogeneous surfaces for the 24 h they were monitored. In highly acidic environments, on the other hand, freshly prepared nanostructures display irregular puncture holes and depressions across their surfaces, indicative of degradation. Complete degradation occurs after 24 h in acidic environments, observed by comparing Figure 3A/F and B/G. Further evidence of degradation is observed in DLS, where trimodal traces with lower hydrodynamic diameters are observed for samples aged 24 h in highly acidic conditions, Figure S14A.^65^ Cratered surface features are not observed for samples produced at neutral or basic conditions, and the intensity average DLS data is consistent over 24 h (Figure S14B and C). We hypothesize that at low pH, either the amide groups or the glycosidic linkages of the glycopolymers are completely hydrolyzed which leads to functional group cleavage and onset of degradation.^66,67^ Similar results for polyacrylamides have been reported by Pei and co-workers, where they determined that molecular weight is relatively stable immediately after transition from primary amine to carboxylic acid.^68^ However, long-term exposure to highly acidic conditions leads to chain scission and a decrease in molecular weight. We expect that the PLA block will also be subject to degradation when held at high pH for longer periods of time.^69^

### Encapsulation Studies

Dye loading and encapsulation efficiency for both hydrophilic and hydrophobic molecules were determined as described in the Experimental Section. Curcumin was chosen as a model hydrophobic molecule. Hydrophobic molecules can be encapsulated into the bilayer shell of vesicles formed by Glc 31:69 and Glc 38:62 and into the core of micelles formed by Glc 66:34, Figure 4A. The DL % and EE % for all formulations are summarized in Table 3. The curcumin-loaded micelles exhibit higher DL % and EE % than the vesicles. Micelles produced from Glc 66:34 yield the highest DL % and EE % at 11.55 and 23.10%, respectively. Vesicles produced from Glc 31:69 yield the lowest DL % and EE % at 4.25 and 8.50%, respectively. In the absence of HBCs, curcumin is completely insoluble in water, and the utilization of HBCs allows for the curcumin to be entrapped within nanoparticles that are then well dispersed within solution, producing a yellow, transparent solution. For our systems, we see that with increasing length of the hydrophilic chain, the DL % and EE % of curcumin dye increase. Similar trends have been reported in the literature, where Dattani et al. showed that lower DL % and slower release of a hydrophobic drug were exhibited for vesicles than micelles.^70^

**Figure 4:**
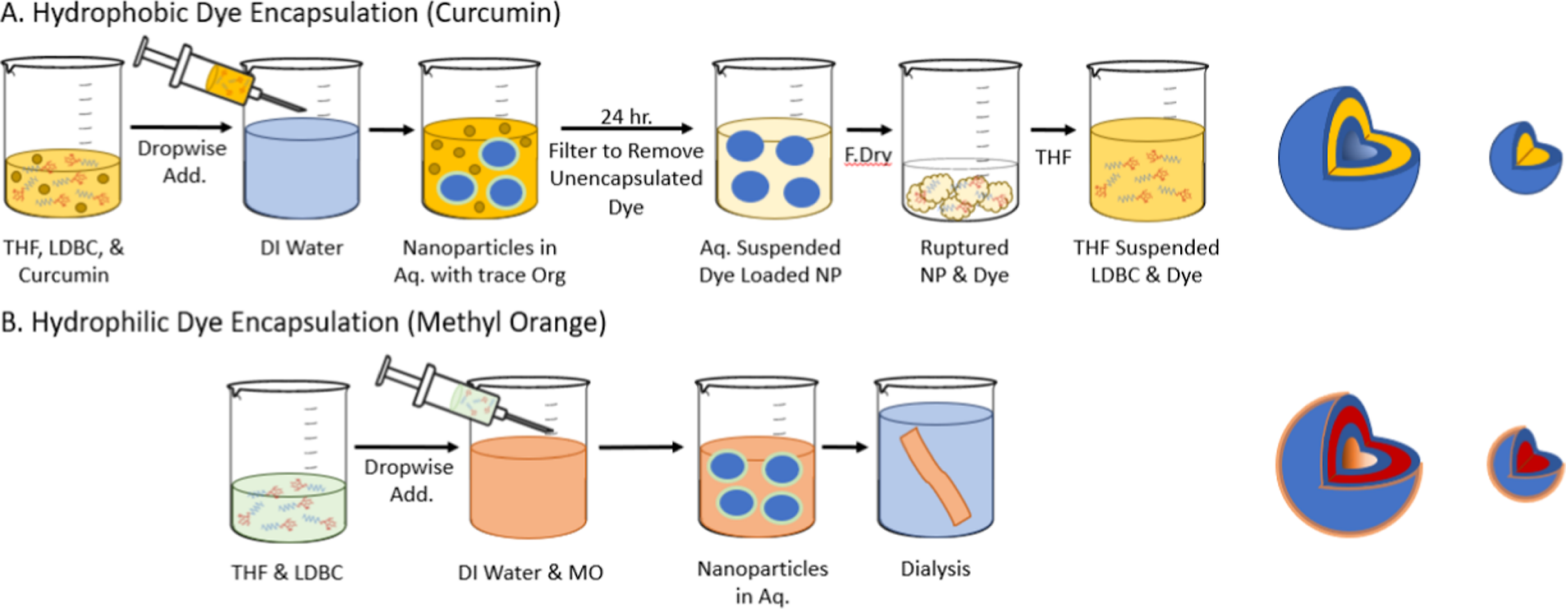
Graphical overview of multistep dye encapsulation accompanied by the proposed structure and dye localization. (A) Hydrophobic dye, curcumin, is introduced in the organic phase and (B) hydrophilic dye, MO, is introduced in the aqueous phase.

**Table 3:** Loading and Encapsulation Efficiencies of Nanostructures with MO and Curcumin Dyes

sample	hydrophobic (curcumin)		hydrophilic (methyl orange)	
	DL (%)	EE (%)	DL (%)	EE (%)
Glc 31:69	4.25	8.50	4.08	2.04
Glc 38:62	5.27	10.34	5.88	2.94
Glc 66:34	11.55	23.10	4.08	2.04

MO was chosen as a model anionically charged, hydrophilic molecule. Nanostructures formed by Glc 31:69 and Glc 38:62 display evidence of bilayer vesicles and offer three different locations for hydrophilic molecule interaction: (1) external surface associations with the glycopolymer shell, (2) internal surface associations within the glycopolymer layer, and (3) entrapment within the interior water pocket, Figure 4B. Solid core–shell micelles formed by Glc 66:34 offer only external surface associations with the glycopolymer shell. Table 3 summarizes the DL % and EE % values for all formulations. Similar DL % and EE % are seen for all three systems, and similar trends have been reported in the literature, without clear explanation.^71^ Niu et al. hypothesized that increased hydrophilic content results in increased stabilization of hydrophilic drug-loaded nanoparticles.^72^ Higher levels of hydrophilic molecule interactions are expected for the larger bilayer vesicles than for the micelles due to their larger diameters and increased avenues for interactions. As the number of block copolymer chains needed to self-assemble into micelles is orders of magnitude lower than that required to form vesicles, it is likely that there are more micelles in the Glc 66:34 sample than there are vesicles in the Glc 31:69 sample.^73^ The greater number of micelles could potentially result in an increased surface area to volume ratio in the micellar samples, providing a greater number of opportunities for hydrophilic molecule interactions in this system that may make up for the additional avenues for hydrophilic molecule interactions available in the vesicles. Based on the nanostructure diameter determined through AFM, the surface area to volume ratio calculated for Glc 31:69 (0.05) is half of that calculated for both Glc 38:62 (0.12) and Glc 66:34 (0.14), making Glc 31:69 a less efficient system.

Overall, our system design is unique with linear hydrophilic and branched hydrophobic chains, which possess specific structure–property dependencies. With a constant hydrophobic molecular weight and architecture, it is possible to observe the impact of hydrophilic chain length on nanostructure properties. If the number of chains used to form each morphology is determined, then it is possible to determine dye entrapped within each structure as opposed to dye entrapped within the system. Further optimization of the entrapment method can be used to increase the DL % and EE %. Additionally, the affinity for the small molecules chosen and their respective blocks may be further optimized in future work. In general, these structures display promise as delivery vehicles that offer the potential for different types of cargo loading.

### Cytotoxicity

**Figure 5:**
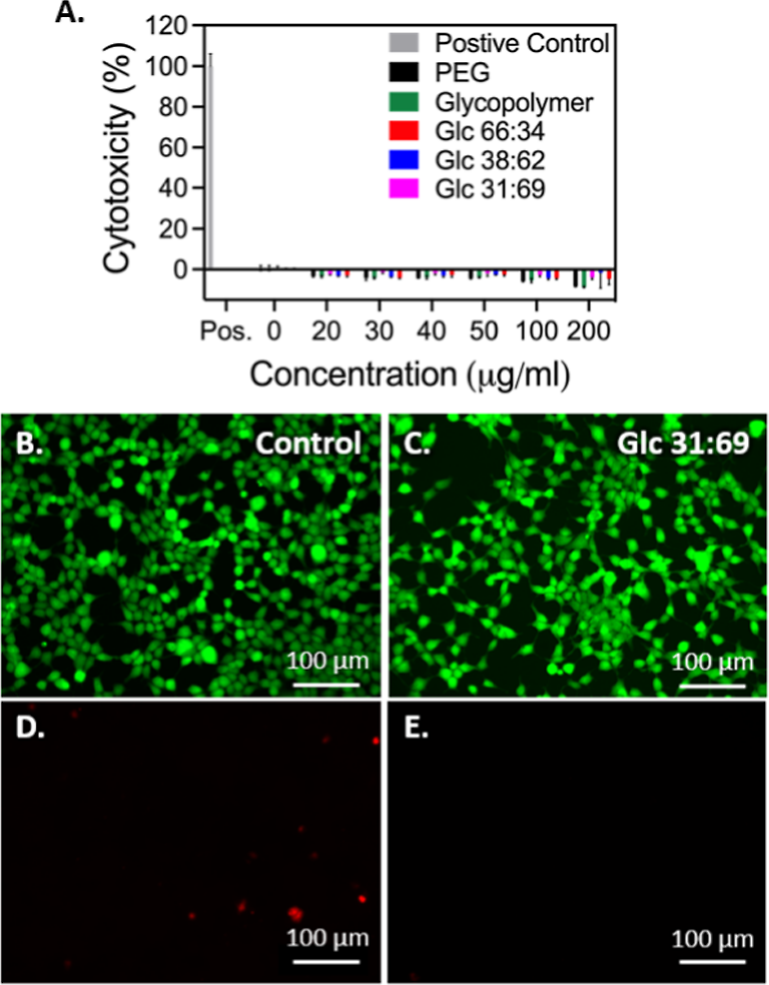
(A) Nanoparticle cytotoxicity after 24 h of exposure in HEK 293 cells determined using an LDH quantification assay. No significant difference exists between PEG and self-assembled glyconanoparticles at all concentrations tested. Data displayed as mean ± s.d. (*n* = 3 per treatment). Analysis of variance with a Tukey post hoc test was used to assess significance (*p* < 0.05). (B–E) Fluorescence imaging of cells treated with 100 μg/mL of the sample for 24 h. (B) and (C) represent live cells stained with calcein-AM. (D) and (E) represent dead cells stained with BOBO-3. Images (B) and (D) are controls and (C) and (E) were incubated with Glc 31:69 nanoparticles. Scale bars are 100 μm.

To determine the cytotoxicity of the self-assembled HBC structures, HEK293 cells were incubated with HBC structures for 24 h followed by quantification of LDH released. PEG, a traditional biocompatible, hydrophilic polymer, was used as a negative control, and Triton X-100 was used as a positive control for comparison in the experiment. As seen in Figure 5, PEG and all glycopolymer formulations have low cytotoxicity (<5%) across all concentrations assayed, with no significant differences between treatments observed. In the previous work, it has been seen that PEG may insert into damaged cell membranes preventing the release of LDH,^74,75^ which, along with the glycopolymers, may result in a slightly reduced LDH release with increasing polymer concentration. As a result, the health of the cells was also validated with the live and dead cell imaging in tandem with the LDH assay at these higher concentrations to ensure cell viability and validity of the data. Representative fluorescent microscopy images following LIVE/DEAD staining (Figures 5 and S16) show little evidence of cell death compared to controls. This data demonstrate the potential of our HBCs to be utilized as biocompatible delivery vehicles for drug and dye delivery.

## Conclusions

This work emphasizes the importance of tailored polymer design in nanomedicine, offering insights into the structure–property relationships of HBCs and their potential as biocompatible delivery vehicles for drug and dye delivery in biomedical research and disease diagnostics. In this study, HBC composed of β-d-glucose pendant glycopolymers and branched PLA were synthesized through thiol–ene photocoupling and investigated for their self-assembled morphology and dye-uptake capability. HHB was varied by changing the chain length of the hydrophilic glycopolymer to understand its impact on nanostructure formation. HBCs with hydrophilic (glycopolymer) ratios of ∼30% yielded bilayer vesicles, those with ∼40% showed a mixture of vesicles and micelles, and those with ∼60% yielded micelles. At hydrophilic content >70%, we observed the formation of unstable aggregates. Stable nanostructures of all compositions (30–60% hydrophilic) demonstrated potential for different cargo-loading abilities, effectively encapsulating both hydrophobic and hydrophilic molecules. Nanoparticles were found to be stable at neutral and basic pH but degraded within 24 h in acidic conditions. Importantly, cytotoxicity assays revealed low toxicity of the HBC structures, indicating their biocompatibility and potential for use in biomedical applications.

Further research in this direction holds promise for advancing noninvasive bioimaging techniques and improving disease diagnostics and therapeutics. Through polymer tailorability, further optimization of HBC design may be used for in vivo targeting or inclusion of stimulus-responsive segments.

## Supporting Information Available

Synthesis and NMR characterization of acetylated glycomonomer (AcGlcEAm), homopolymers (pGlcEAm), and PLA precursor (2,2,5-trimethyl-1,3-dioxane-5-carboxylic acid); polymerization kinetics plots; characterization of homopolymer and block copolymer structures by NMR; UV–vis absorption spectra of glycopolymers; TEM of self-assembled structures; DLS for all compositions and pH-adjusted samples; dye absorbance spectra; and fluorescence imaging of LIVE/DEAD cells (PDF)
