# Supplementary material for: Biocompatible Glycopolymer-PLA Amphiphilic Hybrid Block Copolymers with Unique Self-Assembly, Uptake, and Degradation Properties
Source: Biomacromolecules. 2024 Sep 14;25(10):6681–92. doi: 10.1021/acs.biomac.4c00885 (PMC11480976; doi:10.1021/acs.biomac.4c00885)
Supplement: Supplementary file 1 — bm4c00885_si_001.pdf [file bm4c00885_si_001.pdf]

Supporting Information

# Biocompatible glycopolymer-PLA amphiphilic hybrid block copolymers with unique self-assembly, uptake, and degradation properties

*Kevin A. Green[1], Anuja S. Kulkarni[2,3], Penelope E. Jankoski[1], Thomas B. Newton[1], Blaine Derbigny[2,3], Tristan D. Clemons[1], Davita L. Watkins[2,3*], Sarah E. Morgan[1*]*

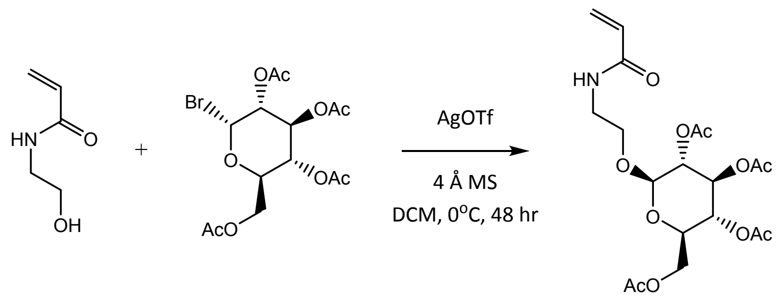


**Scheme S1**. Synthesis of acetal protected glycomonomers, AcGlcEAm.

**1.1 Glycomonomer Synthesis.** The acetyl protected glucose pendant acrylamide monomer, 2′-acrylamidoethyl-2,3,4,6-tetra-O-acetyl-β-D-glucopyranoside (AcGlcEAm), was synthesized following previous literature procedures (Scheme S.1).[1-3] In short, N-hydroxyethyl acrylamide (21.00 g, 182.4 mmol) in excess was added with acetobromo-α-D-glucose (15.00 g, 36.5 mmol) in distilled DCM (400 mL) to a 1000 mL, 3-neck round-bottom flask. Dry molecular sieves (20.00 g, 4 Å size) were added to the reaction mixture to ensure that the reaction was completely dry. The reaction mixture was submerged in an ice bath and allowed to mix for 30 minutes using a mechanical stirrer. At this point, a metal catalyst, silver trifluoromethanesulfonate (14.00 g, 52.2 mmol), was added, and the reaction was allowed to proceed for 48 hours in the dark. The reaction mixture was then filtered, and the crude solution was washed with 1 M HCl (3 x 100 mL), washed with deionized (DI) water (3 x 100 mL), dried over sodium sulfate, and filtered once more. The collected liquid was concentrated through rotary evaporation and purified through column chromatography ($SiO_2$, 10:1 EtOAc:hexanes). Fractions collected after purification were then completely dried through rotary evaporation to afford a white crystalline solid (5.49 g, 33.8%). $^1H$ NMR (400 MHz, $CDCl_3$) δ [ppm] 1.97, 2.03, 2.14 (s, s, s, 12H-13, 14, 15, 16), 3.57 (m, 2H-5), 3.70 (m, 1H-9), 3.91 (m, 2H-4), 4.14 (m, 2H-11, 12), 4.48 (m, 1H-6), 5.02 (d of d, 1H-10), 5.16 (m, 1H-7), 5.38 (t, 1H-8), 5.66 (d of d, 1H-1), 6.05 (m, 1H-2), 6.31 (m, 1H-3).

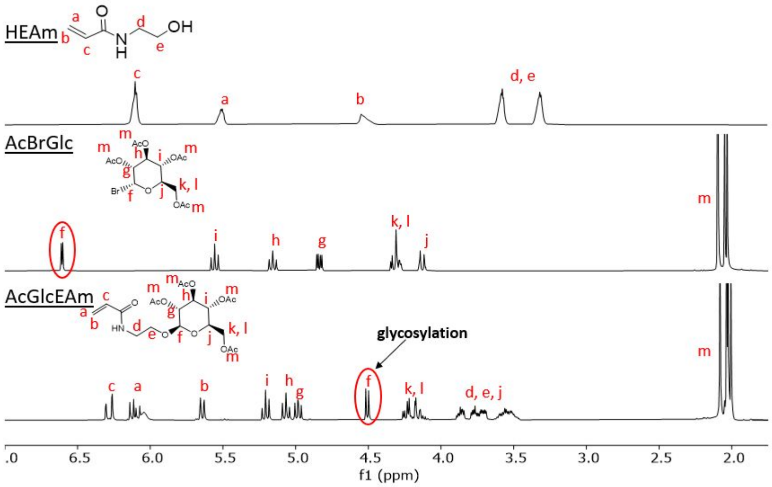


**Figure S1.** $^1$H NMR spectra comparison of HEAm, AcBrGlc, and AcGlcEAm in $CDCl_3$.

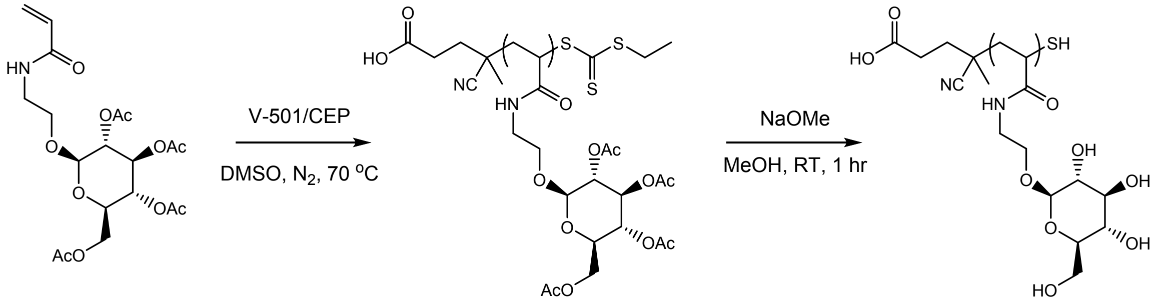


**Scheme S2.** Synthesis of glucose-functionalized glycopolymers via RAFT polymerization and base-catalyzed deprotection.

**1.2 RAFT Polymerization of Glycomonomer.** Glucose functionalized glycopolymers (pGlcEAm) were synthesized similar to previous literature procedures (Scheme S.2).[3] Glycopolymers with a target degree of polymerization (DP) of 20 (molecular weight = 5,870 g/mol), 30 (molecular weight = 8,533 g/mol), and 115 (molecular weight = 31,967 g/mol) were synthesized by RAFT polymerization. The acetyl-protected glycomonomer (AcGlcEAm), chain transfer agent (CEP), thermal initiator (V-501), and internal standard (trimesic acid) were dissolved in anhydrous DMSO in a 25-mL round-bottom flask equipped with a magnetic stirring bar and degassed with ultrapure $N_2$ for 45 minutes while stirring. All polymerizations were carried out at 0.4 M initial monomer concentration. The $[M]_0$:$[CTA]_0$ varied depending on the target molecular weight for each reaction, while the $[CTA]_0$:$[I]_0$ was maintained at 5:1. Reactions were carried out to achieve a final monomer conversion of 60%. To determine reaction times needed to achieve desired molecular weights, kinetic studies were performed where the disappearance of the vinyl monomer peaks (6.0 ppm, 2H and 5.5 ppm, 1H) was monitored by $^1$H NMR spectroscopy (Fig. S.2) and compared to the carboxylic acid peak (8.6 ppm, 3H) of trimesic acid at different reaction times. After the target molecular weights were achieved, the reaction was quenched by submerging the flask into $LN_2$ and precipitating the reaction mixture into DI water. The liquid was decanted leaving behind a yellow solid (pAcGlcEAm). In order to remove both the acetyl protecting groups and the trithiocarbonate end group, residual from CEP,

the polymer was dissolved in methanol (25 mL) and sodium methoxide was added in excess while stirring for 1 hour. The deprotected polymer precipitated from solution, this mixture was centrifuged, and the polymer was then isolated by decanting the remaining liquid. The solid polymer was redissolved in DI water, dialyzed using Spectrum™ Labs Spectra/Por™ 3.5 kDa MWCO standard RC tubing for 3 days in DI water, and lyophilized to isolate the pGlcEAm as a white powder (72% yield). $^1$H NMR (600 MHz, $D_2O$) δ [ppm] 3.49−4.11 (m, 10H-4, 5, 7, 8, 9, 10, 11, 12), 4.39 (d, 1H-6), 5.75 (d of d, 1H-1), 6.19 (m, 1H-3, 1H-2).

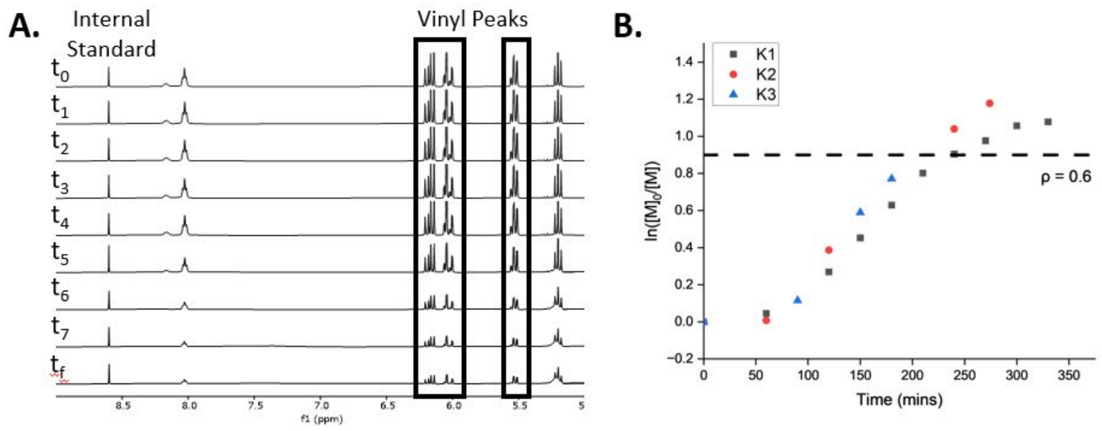


**Figure S2.** (A) $^1$H NMR spectra of the RAFT polymerization of glucose-functionalized glycopolymers tracking conversion over time in DMSO-$d_6$. (B) Plots of ln([M]o/[M]) vs. time for RAFT polymerization of pAcGlcEAm with CEP and V-501 at 70°C in DMSO. The dotted line represents a monomer conversion (ρ) of 60%.

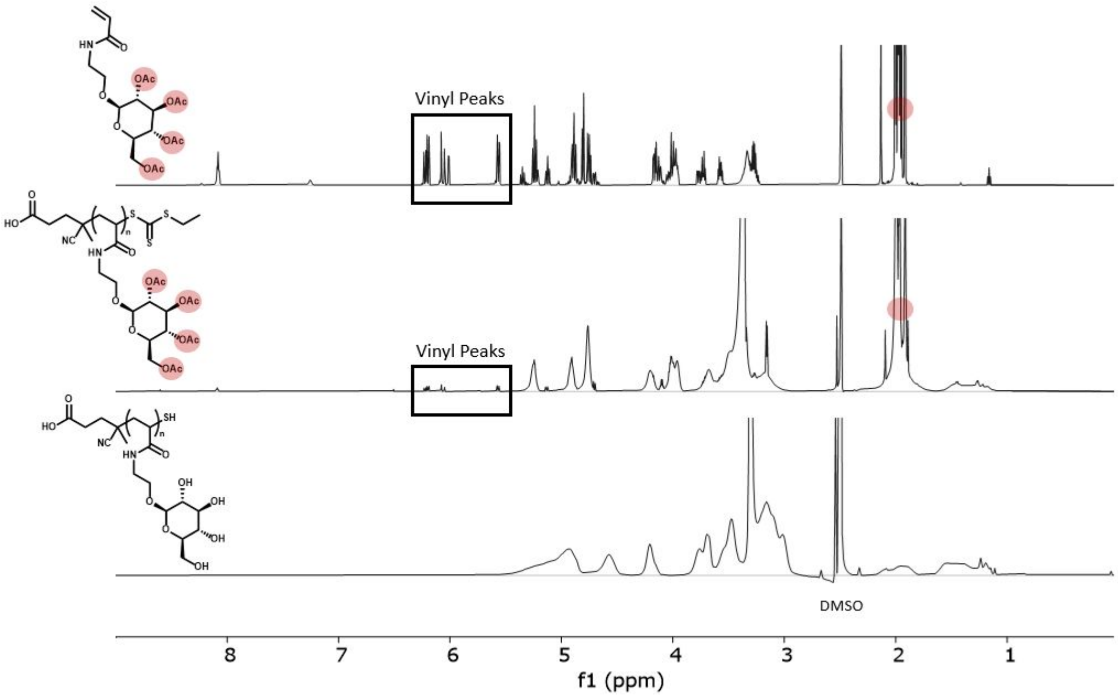


**Figure S3.** $^1$H NMR spectra comparison for AcGlcEAm, pAcGlcEAm, and pGlcEAm in DMSO-$d_6$.

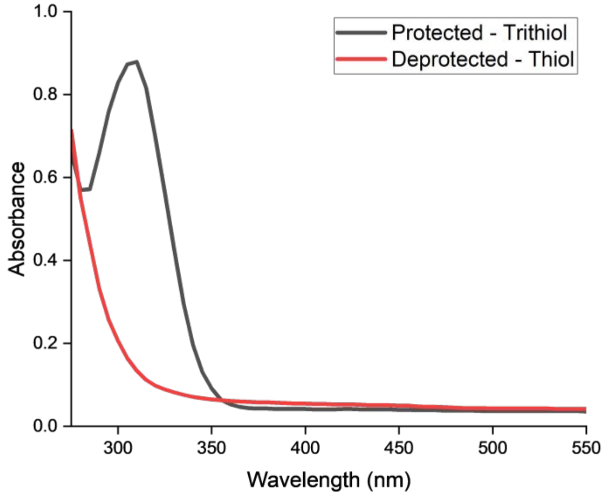


**Figure S4.** UV-Vis absorption spectra of pAcGlcEAm (black) and pGlcEAm (red) in DMSO. The decrease in absorbance at 308 nm indicates cleavage of trithiocarbonate chain ends following base-catalyzed deprotection.

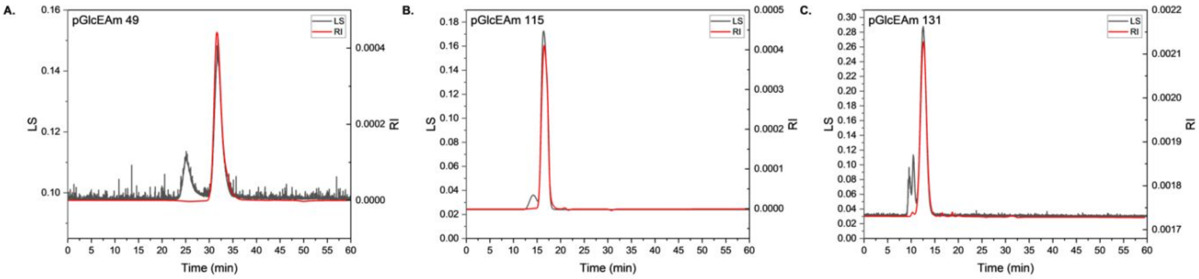


**Figure S5.** ASEC-MALLS trace of A) pGlcEAm 49, B) pGlcEAm 115, and C) pGlcEAm 131 determined in TRIS buffer (pH 8) with 0.01% (w/v) $NaN_3$.

### 1.3 PLA Synthesis

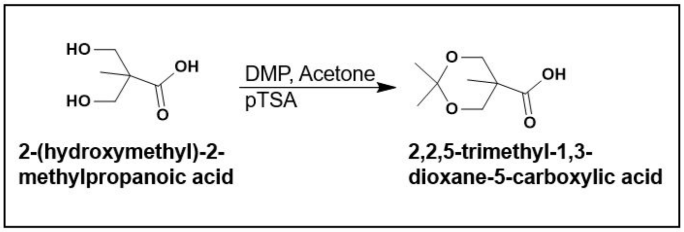


**Scheme S3.** Synthesis of 2,2,5-trimethyl-1,3-dioxane-5-carboxylic acid.

This product was synthesized following a previously reported procedure.[4] Briefly, 2-(hydroxymethyl)-2-methylpropanoic acid (Bis-MPA) (10 g, 75 mmol) was stirred in 50 mL of

acetone, to it 2,2-dimethoxypropane (DMP) (11.6 g, 112 mmol) and PTSA (0.71 g, 4.00 mmol) were added under room temperature and the reaction mixture was allowed to stir for 4 h. Then it was filtered through an amberlyst column, and the eluted solvent was concentrated and was put under 60°C and full vacuum for 2 h to remove the DMP if present. Then, the residue was dissolved in chloroform and precipitated in hexanes, and this procedure was repeated thrice; the precipitate was dried overnight to give (1), a white solid 90% yield (12.01 g). $^1$H NMR (400 MHz, $CDCl_3$) δ [ppm] 4.17 (m, 2H), 3.69 (m, 2H), 1.44 (d, d, 6H, *J=12 Hz and 16 Hz*), 1.20 (s, 3H).

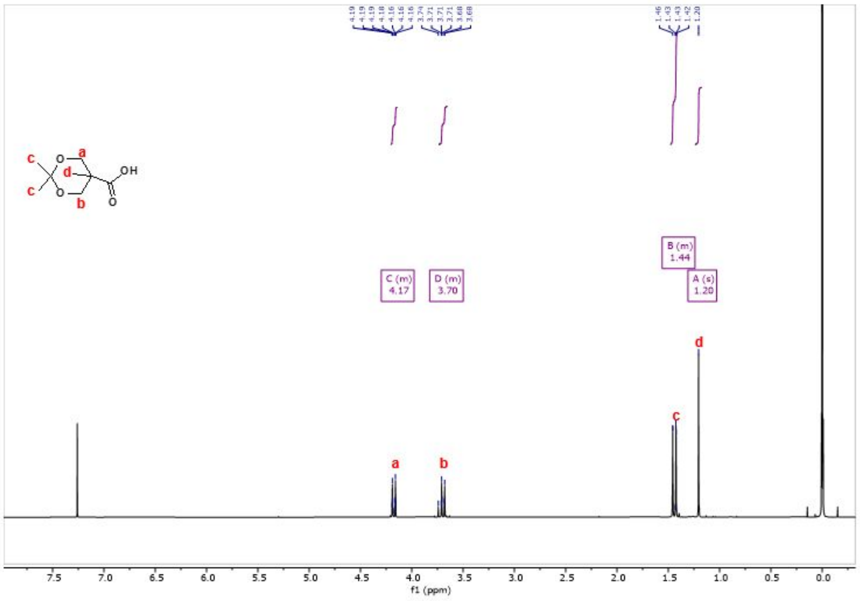


**Figure S6.** $^1$H NMR spectrum of 2,2,5-trimethyl-1,3-dioxane-5-carboxylic acid (**1**) in $CDCl_3$.

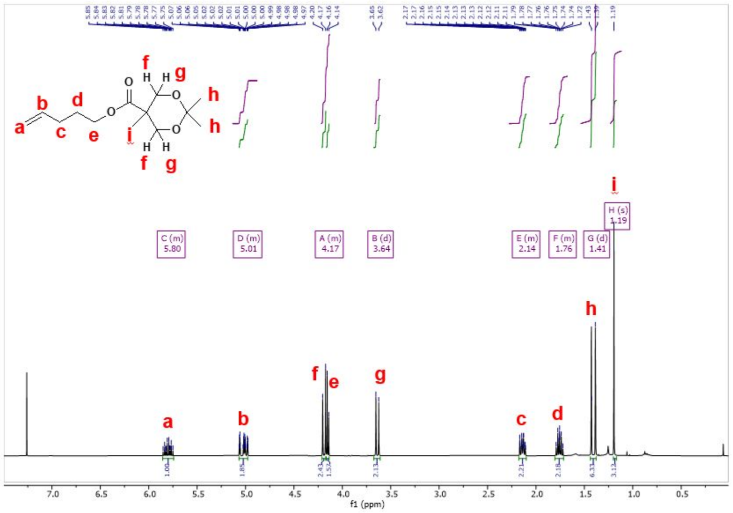


**Figure S7.** $^1$H NMR spectrum of 4-pentene-1-acetonide (**2**) in $CDCl_3$.

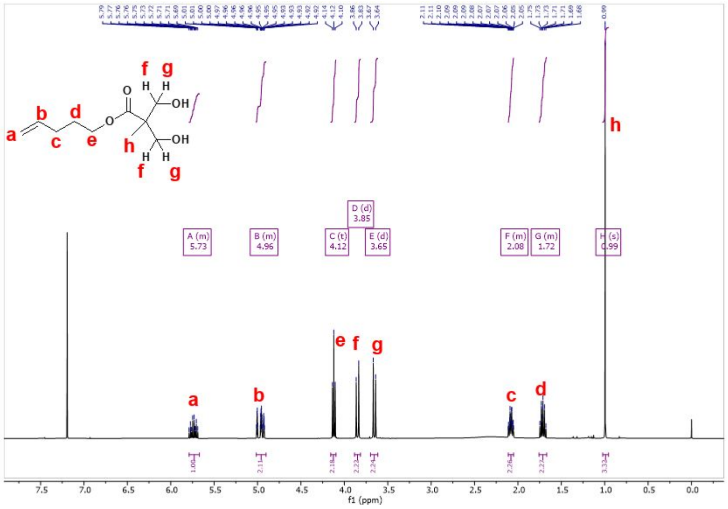

**Figure S8.** $^{1}$H NMR spectrum of 4-pentene-2-hydroxyl (**3**) in $CDCl_3$.

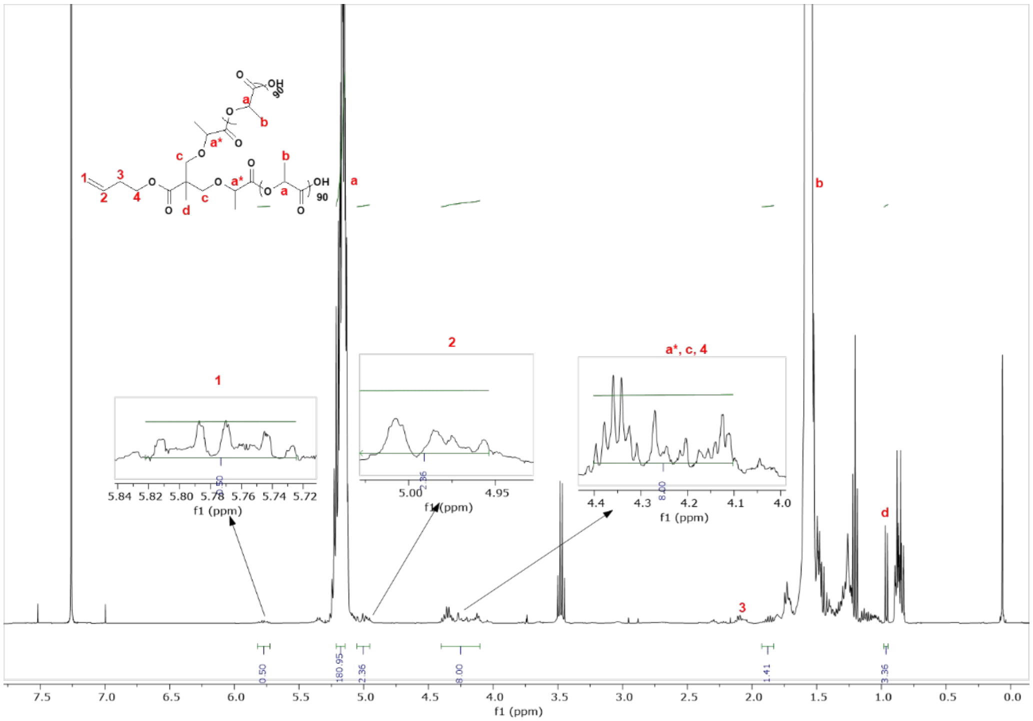

**Figure S9.** $^{1}$H NMR spectrum of branched D, L lactide (**4**) in $CDCl_3$

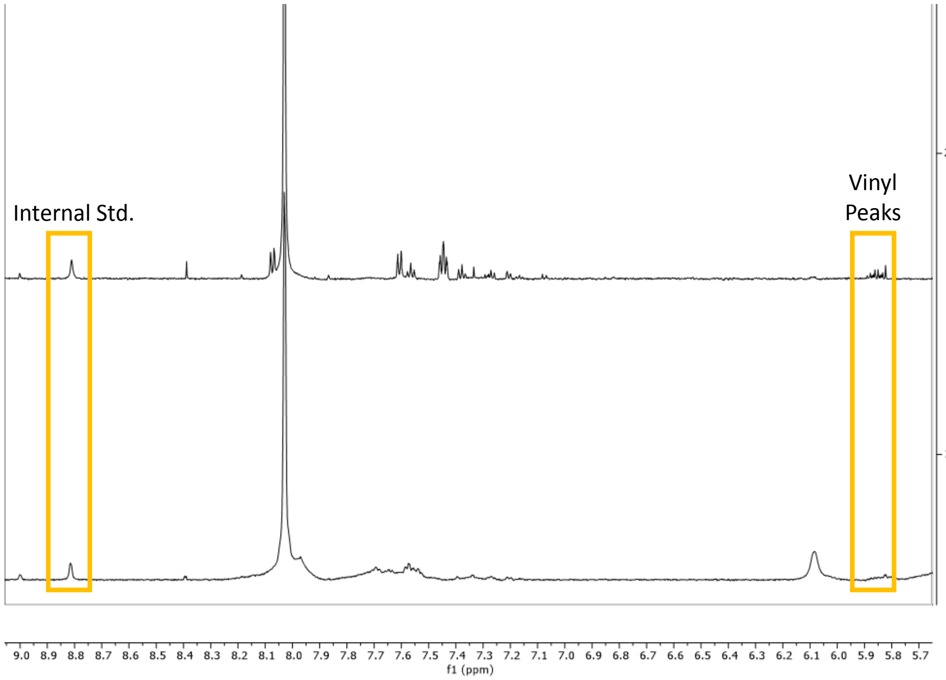


**Figure. S10.** $^1$H NMR spectra of the photocoupling of Glc 31:69 tracking conversion in DMF-$d_7$.

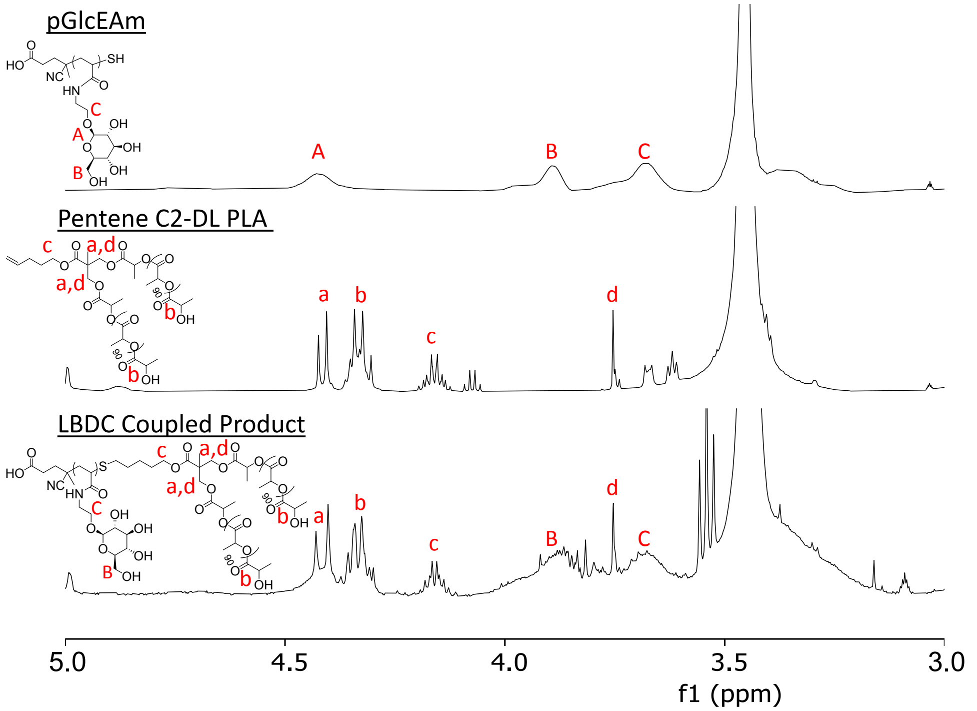


**Figure S11.** $^1$H NMR spectra comparison of pGlcEAm, Pent C2-PLA, and Glc 31:69 coupled LDBC in DMF-$d_7$.

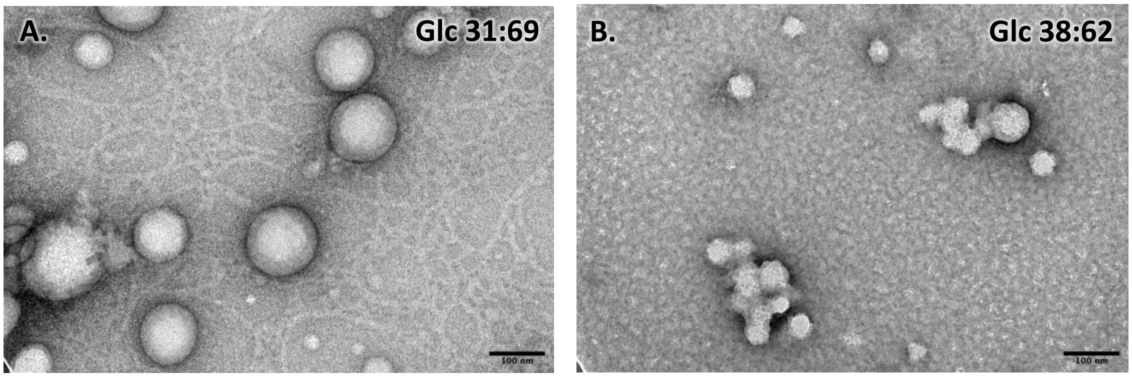


**Figure S12.** TEM images of (A) Glc 31:69 and (B) Glc 38:62. TEMs were collected with uranyl acetate as the contrasting agent.

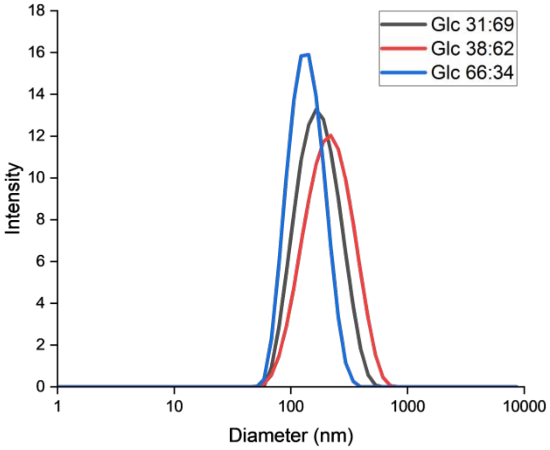


**Figure S13.** DLS intensity average size distribution of nanoparticles formed in water by nanoprecipitation. Glc 31:69 (black), Glc 38:62 (red), and Glc 66:34 (blue).

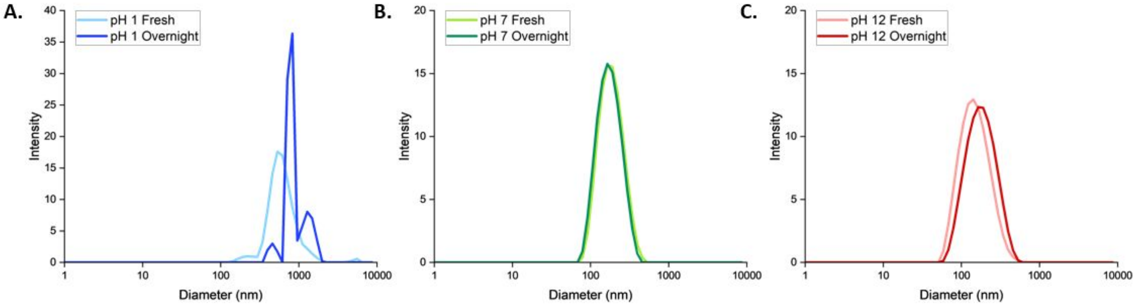


**Figure S14.** DLS intensity average size distribution of nanoparticles formed in varying pH aqueous environments at time of nanoprecipitation and after 24 hrs. A) pH = 1, B) pH = 7, and C) pH = 12.

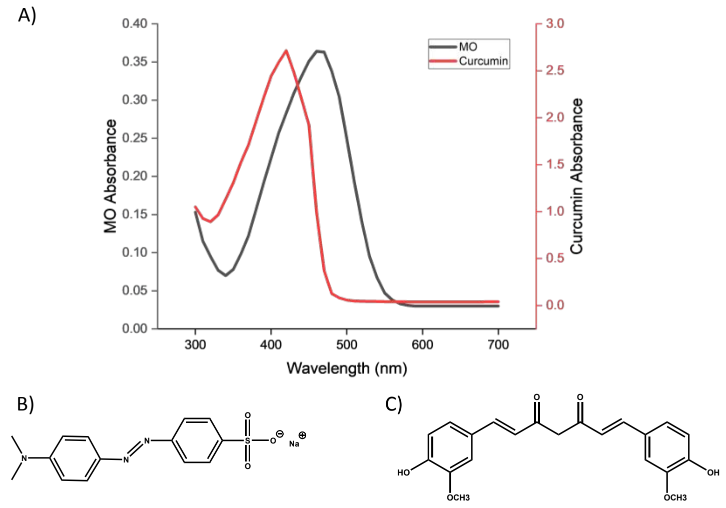


**Figure S15.** A) Absorbance spectra for dyes used in encapsulation studies, B) and C) structures of MO and curcumin. Absorbance spectra of MO and curcumin were performed in water and THF, respectively.

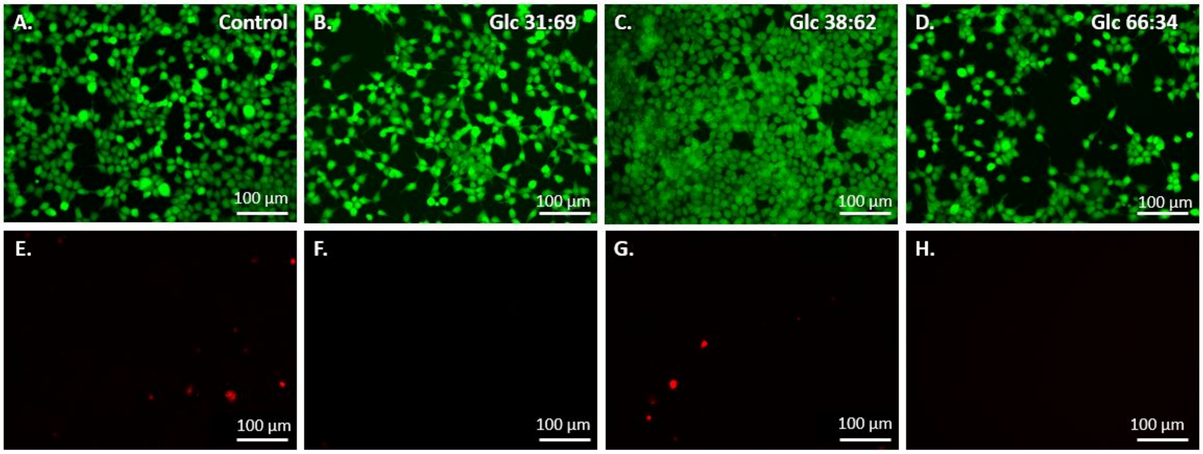


**Figure S16.** Fluorescence imaging of cells treated with 100 µg/mL of sample for 24 hrs. A-D) represent live cells stained with calcein-AM and E-H) represent dead cells stained with BOBO-3. Images A and E) control, B and F) Glc 31:69, C and G) Glc 38:62, and D and H) Glc 66:34. Scale bars are 100 µm.

## 1.4 References


1. Das, P. K.; Dean, D. N.; Fogel, A. L.; Liu, F.; Abel, B. A.; McCormick, C. L.; Kharlampieva, E.; Rangachari, V.; Morgan, S. E., Aqueous RAFT Synthesis of Glycopolymers for Determination of Saccharide Structure and Concentration Effects on Amyloid beta Aggregation. *Biomacromolecules* **2017,** *18* (10), 3359-3366.
2. Bristol, A. N.; Saha, J.; George, H. E.; Das, P. K.; Kemp, L. K.; Jarrett, W. L.; Rangachari, V.; Morgan, S. E., Effects of Stereochemistry and Hydrogen Bonding on Glycopolymer-Amyloid-beta Interactions. *Biomacromolecules* **2020,** *21* (10), 4280-4293.
3. Stockmal, K. A.; Downs, L. P.; Davis, A. N.; Kemp, L. K.; Karim, S.; Morgan, S. E., Cationic Glycopolyelectrolytes for RNA Interference in Tick Cells. *Biomacromolecules* **2022,** *23* (1), 34-46.
4. Yaddehige, M. L.; Chandrasiri, I.; Barker, A.; Kotha, A. K.; Dal Williams, J. S.; Simms, B.; Kucheryavy, P.; Abebe, D. G.; Chougule, M. B.; Watkins, D. L., Structural and Surface Properties of Polyamidoamine (PAMAM) – Fatty Acid-based Nanoaggregates Derived from Self-assembling Janus Dendrimers. *ChemNanoMat* **2020,** *6* (12), 1833-1842.
